# The Microbiological Profile and Antibiotic Susceptibility of Fracture Related Infections in a Low Resource Setting Differ from High Resource Settings: A Cohort Study from Cameroon

**DOI:** 10.3390/antibiotics13030236

**Published:** 2024-03-04

**Authors:** Loïc Fonkoue, Elizabeth K. Tissingh, Michelle Tognia Ngouateu, Kennedy Olivier Muluem, Olivier Ngongang, Pretty Mbouyap, Perrin Ngougni Pokem, Kuetche Fotsing, Jean Bahebeck, Martin McNally, Olivier Cornu

**Affiliations:** 1 Department of Orthopedics and Trauma, Yaoundé General Hospital, Yaounde P.O. Box 5408, Cameroon; kenedymuluem@yahoo.fr; 2 Department of Surgery and Specialties, University of Yaounde 1, Yaounde P.O. Box 1364, Cameroon; bantouboy@gmail.com (O.N.); jbahebeck@yahoo.com (J.B.); 3 Experimental and Clinical Research Institute, Université Catholique de Louvain, 1200 Brussels, Belgium; olivier.cornu@saintluc.uclouvain.be; 4 Royal National Orthopedic Hospital NHS TRUST, London HA7 4LP, UK; elizabeth.tissingh1@nhs.net; 5 King’s Global Health Partnerships, School of Life Course and Population Sciences, King’s College London, London SE1 1UL, UK; 6 Department of Microbiology, Université des Montagnes, Bangante P.O. Box 208, Cameroon; michellengouateu@gmail.com (M.T.N.); kuetchefotsing@gmail.com (K.F.); 7 Department of Microbiology, Centre Pasteur du Cameroun, Yaoundé P.O. Box 1274, Cameroon; prmbouyap@yahoo.fr; 8 Pharmacologie Cellulaire et Moléculaire, Louvain Drug Research Institute, Université Catholique de Louvain, 1200 Brussels, Belgium; perrin.ngougni@uclouvain.be; 9 Department of Microbiology, Cliniques Universitaires Saint-Luc, Université Catholique de Louvain, 1200 Brussels, Belgium; 10 Bone Infection Unit, Nuffield Orthopaedic Centre, Oxford University Hospitals, Oxford OX3 7HE, UK; bonemcn@gmail.com; 11 Department of Orthopedics and Trauma, Cliniques Universitaires Saint-Luc, 1200 Brussels, Belgium

**Keywords:** fracture-related infection, microorganisms, antibiotic resistance, low-resource setting

## Abstract

Fracture-related infection (FRI) is a common and devastating complication of orthopedic trauma in all settings. Data on the microbiological profile and susceptibility of FRI to antibiotics in low-income countries are scarce. Therefore, this study aimed to investigate the microbial patterns and antimicrobial susceptibility of FRI in a sub-Saharan African setting in order to provide guidance for the formulation of evidence-based empirical antimicrobial regimens. We conducted a retrospective analysis of patients treated for FRI with deep tissue sampling for microbiological culture from January 2016 to August 2023 in four tertiary-level hospitals in Yaoundé, Cameroon. There were 246 infection episodes in 217 patients. Cultures were positive in 209 (84.9%) cases and polymicrobial in 109 (44.3%) cases. A total of 363 microorganisms from 71 different species were identified, of which 239 (65.8%) were Gram-negative. The most commonly isolated pathogens were *Staphylococcus aureus* (n = 69; 19%), *Enterobacter cloacae* (n = 43; 11.8%), *Klebsiella pneumoniae* (n = 35; 9.6%), *Escherichia coli* (n = 35; 9.6%), and *Pseudomonas aeruginosa* (n = 27; 7.4%). *Coagulase-negative staphylococci* (CoNS) were isolated in only 21 (5.9%) cases. Gram-negative bacteria accounted for the majority of the infections in early (70.9%) and delayed (73.2%) FRI, but Gram-positive bacteria were prevalent in late FRI (51.7%) (*p* < 0.001). Polymicrobial infections were more frequent in the early (55.9%) and delayed (41.9%) groups than in the late group (27.6%) (*p* < 0.001). Apart from *Staphylococcus aureus*, there was no significant difference in the proportions of causative pathogens between early, delayed, and late FRI. This study found striking resistance rates of bacteria to commonly used antibiotics. MRSA accounted for 63% of cases. The most effective antibiotics for all Gram-positive bacteria were linezolid (96.4%), vancomycin (92.5%), clindamycin (85.3%), and fucidic acid (89.4%). For Gram-negative bacteria, only three antibiotics displayed a sensitivity >50%: amikacin (80.4%), imipenem (74.4%), and piperacillin + tazobactam (57%). The most effective empirical antibiotic therapy (with local availability) was the combination of vancomycin and amikacin or vancomycin and imipenem. In contrast to the literature from high-resource settings, this study revealed that in a sub-Saharan African context, Gram-negative bacteria are the most common causative microorganisms of FRI. This study revealed striking resistance rates to commonly used antibiotics, which will require urgent action to prevent antimicrobial resistance in low and middle-income countries.

## 1. Introduction

Fracture-related infection (FRI) is a major global concern in orthopedic trauma. It is a devastating complication of trauma and trauma surgery, which may require repeated surgical procedures, long-term antimicrobial therapy, and prolonged bone healing time and often leads to poor functional outcomes [1,2]. It is associated with increased socioeconomic costs, morbidity, and mortality. It is a common complication, with a prevalence of 1–2% after internal fixation of closed fractures and up to 30% in open fractures, making prevention and treatment strategies a priority [1]. Knowledge of the microbial epidemiology of FRI is mandatory to provide optimal local or systemic antimicrobial treatment, especially for empirically treated cases or when culture results are negative [3]. Recommendations concerning the prevention and treatment of FRI have been recently proposed, and empirical antimicrobial therapy (EAT) regimens have been adopted based on microbial epidemiology [3,4,5,6,7].

Although there has been an increase in FRI publications since the consensus definition publication in 2018 [8], data from low and middle-income countries (LMIC) are scarce. Yet the burden of FRI in LMIC is higher than in high-income countries (HIC), with rates up to 4.1–10% after internal fixation of closed fractures and up to 51.2% in open fractures [9,10,11]. Data on the detailed analysis of pathogens found in FRI from LMIC and their antibiotic susceptibility are limited. There are recommendations from HIC that EAT for FRI should be broad-spectrum, including a lipopeptide or a glycopeptide and an agent covering Gram-negative bacilli [5]. As there is no evidence-based guideline from LMIC, EAT protocols in those settings are often extrapolated from the results of the studies conducted in HIC, where the conditions may be very different [12]. Due to different national, geographical, and socioeconomic conditions, the bacterial epidemiology might be different, leading to different EAT recommendations [12]. Identifying the microbiological pathogens responsible for FRI in that specific environment is necessary to select appropriate initial antibiotic therapy and develop adequate prevention strategies. This is especially relevant for low-resource settings where the infrastructure needed to identify causative microorganisms may be lacking or inadequate, or if present, many patients cannot afford a microbial culture as financial means are limited.

The current limited literature on the microbial profile of FRI in LMICs shows certain trends that need to be confirmed. Although *Staphylococcus aureus* is the most common organism, polymicrobial infections are more frequent, and Gram-negative pathogens seem to be more prevalent than in HICs [13,14,15]. In addition, antimicrobial resistance is of particular concern, requiring rigorous epidemiological surveillance and appropriate action [16].

There has been a debate in the recent literature regarding the relationship between the Willenegger and Roth FRI classification [17] and the microbiological etiology of FRI, which could guide EAT [6,18,19,20]. This differentiation between early, delayed, and late may be less relevant in low-resource settings, particularly because patients often present late, the time frame can be unclear, and there is a general overuse of antibiotics.

The aim of this study was to investigate the microbial patterns and antimicrobial susceptibility of FRI in a sub-Saharan African setting in order to provide guidance for the formulation of evidence-based empirical antimicrobial regimens.

## 2. Results

Over the study period, 224 patients with FRI fulfilled the inclusion criteria. Seven cases were excluded for spine infection and incomplete medical files, and 217 patients were retained for analysis for a total of 246 infectious episodes. There were 150 (69.1%) men and 67 (30.9%) women, with a mean age of 40.6 ± 15.1 years. The most frequent sites of FRI were the tibia and femur, with 95 (42.6%) and 87 (39.0%) cases, respectively, and open fractures were responsible for 107 (47.9%) cases (Table 1). Of the 246 microbiological cultures, 209 (84.9%) were positive. A total of 363 organisms were isolated, made up of 71 distinct species. Infection was presented early in 115 (53.0%) cases, delayed in 37 (17.1%) cases, and late in 65 (29.9%) cases. Infection was polymicrobial in 109 (44.3%) cases, monomicrobial in 100 (40.6%) cases, and culture-negative in 37 (15.1%).

### 2.1. Profile of Isolated Microorganisms

Table 2 gives a full breakdown of the isolated organisms. The most commonly isolated pathogens were *Staphylococcus aureus* (n = 69; 19%), *Enterobacter cloacae* (n = 43; 11.8%), *Klebsiella pneumoniae* (n = 35; 9.6%), *Escherichia coli* (n = 35; 9.6%), and *Pseudomonas aeruginosa* (n = 27; 7.4%). Gram-positive bacteria (*Staphylococcus aureus*, *coagulase-negative Staphylococcus*, *Streptococcus species*, and *Enterococcus species*) were isolated in 124 (34.2%) cases. *Coagulase-negative Staphylococcus* (CoNS) was isolated in only 21 (5.9%) cases. *Staphylococcus aureus* was significantly more isolated in late infections (33.3%) than in delayed (12.5%) and early (15%) infections (*p* < 0.001) (Table 3). For all other pathogens, there was no significant difference in proportion between early, delayed, and late FRI.

Gram-negative bacteria accounted for the majority of the infections in early (70.9%) and delayed (73.2%) FRI (*p* < 0.001), but Gram-positive bacteria were prevalent in late FRI (51.7%) compared to early (29.1%) and delayed (26.8%) FRI (*p* < 0.001). *Enterobacterales* were isolated in 188 (51.7%) cases, with *Enterobacter cloacae* being the most common pathogen. *Pseudomonas aeruginosa*, the most prevalent microorganism among non-fermenting Gram-negative bacteria, did not show any significant proportional difference between the three groups. Overall, the type of culture results (negative, monomicrobial, and polymicrobial) were significantly different between the early, delayed, and late infection groups. The culture tended to be more polymicrobial in the early (55.9%) and delayed (41.9%) groups than in the late group (27.6%) (*p* < 0.001). In contrast, culture negativity was more frequent in the late group (23.7%) than in the early (8.7%) and delayed (18.6%) groups (*p* = 0.012).

### 2.2. Antimicrobial Susceptibility of Isolated Pathogens

**Figure 1:**
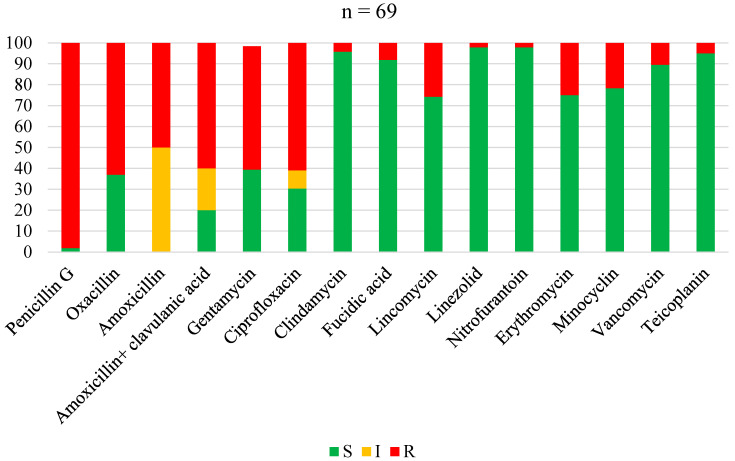
Antibiotic sensitivity and resistance rates of *Staphylococcus aureus* in FRI. S: susceptible; I: susceptible, increased exposure; R: resistant.

**Figure 2:**
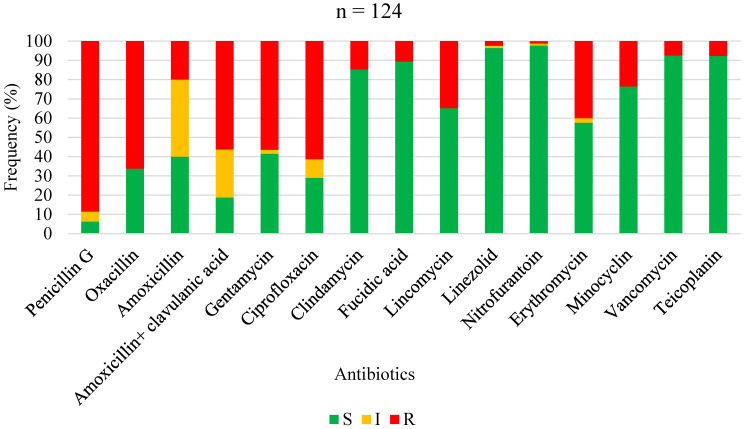
Antibiotic sensitivity and resistance rates of all Gram-positive bacteria in FRI. S: susceptible; I: susceptible, increased exposure; R: resistant.

**Figure 3:**
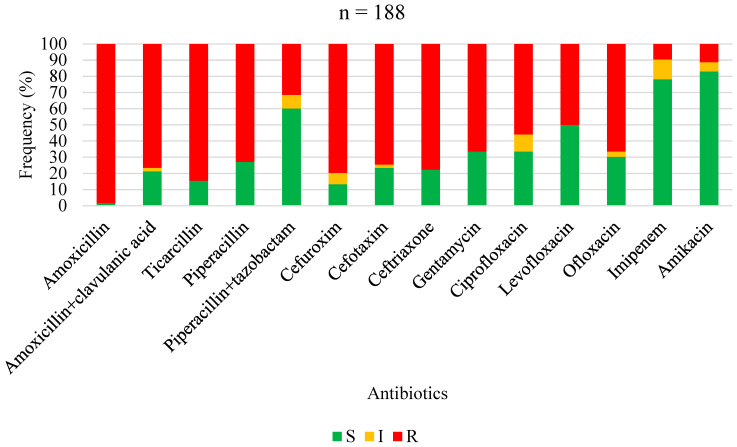
Antibiotic sensitivity and resistance rates of *Enterobacterales* in FRI. S: susceptible; I: susceptible, increased exposure; R: resistant.

**Figure 4:**
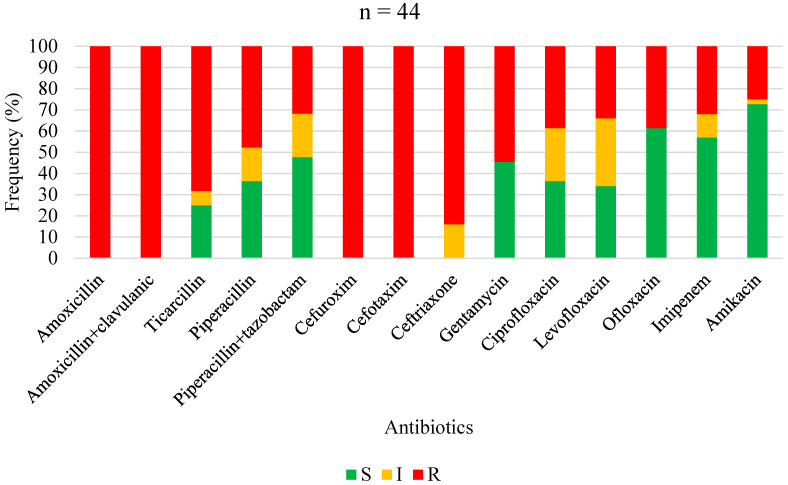
Antibiotic sensitivity and resistance rates of *non-fermenting Gram-negative bacilli* in FRI. S: susceptible; I: susceptible, increased exposure; R: resistant.

**Figure 5:**
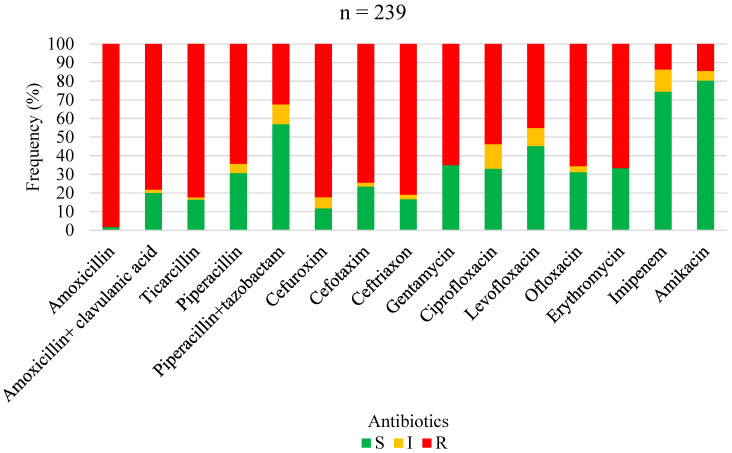
Antibiotic sensitivity and resistance rates of all Gram-negative bacteria in FRI S: susceptible; I: susceptible, increased exposure; R: resistant.

The antimicrobial susceptibility profiles of *Staphylococcus aureus*, overall Gram-positive bacteria, Enterobacterales, non-fermenting Gram-negative bacilli, and overall Gram-negative bacteria are shown in Figure 1, Figure 2, Figure 3, Figure 4 and Figure 5, respectively. Forty-three (62.3%) of the 69 *Staphylococcus aureus* isolates were found to be *Methicillin-resistant* (MRSA). *Staphylococcus aureus* was resistant to ciprofloxacin and levofloxacin in 70% and 76.1% of cases, respectively (Figure 1). The most effective antibiotics for *Staphylococcus aureus* were linezolid (97.9%), teicoplanin (95%), clindamycin (95.8%), fucidic acid (91.9), and vancomycin (89.5%). The sensitivity of *Staphylococcus aureus* to rifampicin was not routinely tested since it is reserved for tuberculosis treatment in our setting. Considering all Gram-positive bacteria (Figure 2), the most sensitive antibiotics were linezolid (96.4%), vancomycin (92.5%), teicoplanin (92.3%), fucidic acid (89.4%), and clindamycin (85.3%). For all Gram-negative bacteria (Figure 5), there were only three antibiotics with a sensitivity >50% in this study: amikacin (80.4%), imipenem (74.4%), and piperacilline + tazobactam (57%). Colimycine and tigecycline were not routinely tested in this study, as these antibiotics are not yet available in most of the LICs. There was a strikingly high resistance rate of Gram-negative bacteria to all easily available and affordable antibiotics in our setting: amoxicillin + clavulanic acid (79.9%), ceftriaxone (83.3%), cefotaxim (76.5%), cefuroxime (88.2%), gentamicin (65%), ofloxacin (68.8%), ciprofloxacin (67%), and levofloxacin (54.8%).

## 3. Discussion

This study investigated the microbiological patterns and antimicrobial susceptibility of pathogens causing FRI in a sub-Saharan African setting in order to assess whether the guidelines for empirical antibiotic therapy from other countries would be effective in our setting. We found that *S. aureus* was the most isolated microorganism (19%), but overall, Gram-negative bacteria represented 65.8% of the isolates. CoNS represented only 5.7% of isolates. In early and delayed-onset infections, Gram-negative bacteria were more prevalent (70.9% and 73.2%, respectively) than in late infections, where Gram-positive bacteria were mostly isolated (51.7%). Apart from *Staphylococcus aureus*, there was no significant difference in the proportions of causative pathogens between early, delayed, and late FRI. This is similar to findings from studies in HICs suggesting that dividing FRIs by the duration of symptoms may not be helpful [18,19,20].

The microbiological profile of FRI found in this study showed important differences with that of studies from HICs. Although *S. aureus* was the most isolated germ (19%), its prevalence was lower than in Europe (Baertl et al., 40.6% [19]; Depypere et al., 31.4% [18]; Patel et al., 34.2% [3]). In contrast, our overall incidence of Gram-negative bacteria (GNB) (65.8%) is two to three times higher than other studies from the HIC [3,18,19,20] (Table 4). However, recent studies in China and South Africa found that GNB represented 47–70% of the causative pathogens of FRI, which is similar to our findings [12,13,15,21]. In these studies, the most commonly isolated pathogens were similar to our findings: *S. aureus*, followed by *Enterobacter cloacae*, *Escherichia coli*, *Acinetobacter baumanii*, and *Pseudomonas aeruginosa* [21]. In contrast, the most commonly isolated pathogens in HICs are *S. aureus*, *CoNS*, *Pseudomonas aeruginosa*, and *Enterobacter species* [3]. The proportion of polymicrobial infections in our study was higher (44.3%) compared to other studies (Depypere et al., 25.3%; Patel et al., 34.2%; Corrigan et al., 36.0%) [3,18,20]. Our results are close to those of two studies that have shown that in the LMICs, polymicrobial infections are frequent and Gram-negative bacteria are the most common in post-traumatic osteomyelitis [13,14]. We believe that these differences could be explained by the fact that early FRI represented 51.6% of all cases in our setting. As our results showed that early FRI was significantly more likely to be polymicrobial, it seems logical that the proportion of polymicrobial infections in this study was high. In addition, open fractures accounted for 47.9% of cases. Due to the delay in the management of open fractures in our setting, leading to high rates of infection, the wound is likely to be infected by multiple hospital-acquired microorganisms before surgery [11]. The higher proportion of early FRI in this resource-limited setting may also be due to poor-quality operating conditions. This may explain why causative pathogens are mainly virulent Gram-negative Enterobacterales, which are known to be responsible for severe hospital-acquired infections.

### NR Not Reported

The antimicrobial susceptibility profile displayed striking rates of resistance to commonly available and affordable antibiotics and a worrying increase in bacterial resistance to the recommended empirical antibiotic therapy. Fluoroquinolones and rifampicin are described as the cornerstones for the treatment of implant-related infections in HICs [5,22]. However, in this study, the resistance rate of Gram-negative bacteria to fluoroquinolones was higher than 60%, and rifampicin is not available as monotherapy in these settings because it is only used in a fixed combination for the treatment of tuberculosis. For Gram-positive bacteria, linezolid, vancomycin, teicoplanin, and fucidic acid were the only antibiotics with 90% (or above) sensitivity. Among these antibiotics, only vancomycin and fucidic acid are commonly available in this country, but they are expensive, limiting their access to patients, given that all the costs are borne by the patient. Fucidic acid and clindamycin could be relevant for oral routes, given that they maintain sensitivity rates above 80%. For Gram-negative bacteria, which are the most frequent, amikacin, imipenem, and piperacillin + tazobactam are the only antibiotics maintaining a sensitivity rate of more than 50%.

There is a very rapid emergence of resistance to these relatively recent antibiotics in our setting, which is striking. A sensitivity rate of all bacteria of only 74% to carbapenems is concerning, and healthcare policies on a global scale must be reviewed to address this alarming situation [23]. A recent study in HICs found a susceptibility rate of 96% to carbapenemes (versus 74% in our study), 100% to vancomycin (versus 92% in our study), and 75% to piperacillin + tazobactam (versus 57%) [18]. Based on the current study, the most effective empirical antibiotic therapy (with available antibiotics) is the combination of vancomycin and imipenem, or vancomycin and amikacin, which would be effective in about 80% of episodes. Not only is this coverage rate low, but also this therapeutic protocol is unaffordable for most patients in LICs, especially since it is a long-term treatment. The average daily cost of this treatment for an adult is approximately USD 78, which is higher than the guaranteed minimum wage in Cameroon (USD 60 per month). Therefore, antimicrobial stewardship in orthopedic infections, as well as education of patients and prescribers on the consequences of inappropriate antimicrobial use, must be the priorities in these countries [24]. In addition, emphasis should be placed on thorough surgical debridement and the need for developing other therapeutic approaches (local antibiotics, phages, local antiseptics, and new antimicrobial agents) to effectively treat these multidrug-resistant bacteria-induced FRIs in low-resource settings. A recent study in the UK recommended a glycopeptide with carbapenem (vancomycin + meropenem) as a systemic empirical antibiotic treatment or a glycopeptide + aminoglycoside (vancomycin + gentamicin) locally [7]. In the current study, we observed a very high resistance rate to gentamicin (65%), so it would not be good systemically, unlike amikacin, whose sensitivity rates were among the highest in this series. However, local gentamicin may still be possible as elution levels are very high, above usual systemic levels, so it would kill many intermediate or even higher-level resistant Gram-negatives, as shown by Bezstarosti et al. [25]. Only very resistant pathogens will not be killed by high-level local gentamicin [25]. Therefore, systemic vancomycin with local gentamicin might still be a cost-effective option.

The retrospective design of this study is a limitation, which did not allow us to correlate the clinical presentation, severity of the cases, treatment, and outcome to microbiological epidemiology and the antibiotic susceptibility profile. In addition, our results reflect our local population, and the susceptibility pattern cannot be generalized without further studies. Moreover, multiple tests have been used in statistics. Nevertheless, to the best of our knowledge, this is one of the first large studies on the microbiology of FRI in a sub-Saharan African context since the publication of the consensus definition of FRI. We believe this study would contribute to the development of guidelines for empirical antibiotic therapy in the management of FRI in a sub-Saharan African context.

## 4. Materials and Methods

### 4.1. Study Design and Patient Identification

This retrospective cohort study reviewed the prospectively collected microbial cultures of patients with FRI from January 2016 to August 2023 in four tertiary-level hospitals in Yaoundé, Cameroon. This study was approved by the ethics committee of the University of Yaoundé 1, Cameroon.

As no standard diagnostic criteria for FRI existed before 2018, all patients with the diagnosis of post-traumatic osteomyelitis, internal fixation-associated infection, or infected fracture were considered. From 2019, FRI was defined based on the definition of the FRI consensus group [8]. We included patients with at least one of the confirmatory diagnostic criteria of FRI, having microbiological culture results from intraoperatively collected tissue samples during the study period. Patients were excluded if FRI affected the skull or spine.

### 4.2. Data Collection

We recorded patient demographics, site of FRI, time of infection, causative pathogens, and susceptibility to antimicrobial agents. FRI were classified into early (infection presenting 0–2 weeks after surgery for closed fractures or after the trauma for open fractures), delayed (presenting at 3–10 weeks), and late (>10 weeks) [17]. The microbiological profile of all the FRI was analyzed, as well as their susceptibility to antimicrobial agents. Mono- and polymicrobial infections were determined. Polymicrobial FRI was defined as more than one microorganism isolated from two or more cultures of deep tissue biopsies.

### 4.3. Microbiological Analysis

Deep biopsies from bone and surrounding tissues were sent for microbiological analysis, performed by the National Referral Laboratory (The Pasteur’s Center), using appropriate media and methods. Tissue specimens were cultured for 7–10 days at 35 °C on aerobic and anaerobic blood agar as well as in thioglycolate broth. Matrix-assisted laser desorption ionization time-of-flight mass spectrometry (Maldi-TOF) was used for identification. Susceptibility to antibiotics was tested on VITEK 2 and interpreted according to European Committee on Antimicrobial Susceptibility Testing (EUCAST) breakpoints (www.eucast.org, last accessed on 21 October 2023). Sonication of any explanted implant and histological examination were not routinely performed during the study period. If the culture results were negative, a polymerase chain reaction (PCR) was generally not performed. Standard microbiological techniques were used to identify microorganisms and determine their susceptibility to antimicrobials [26]. Two or more positive cultures with identical pathogens were considered confirmatory for infection [27]. A single positive culture was also considered when the isolate was a virulent pathogen [18,26] if the diagnosis of FRI was made on other non-microbiological criteria. Virulent pathogens were defined a priori as Gram-negative bacilli, *Staphylococcus aureus*, *Enterococci*, *beta-hemolytic Streptococci*, *Streptococcus pneumoniae,* and *Candida albicans* [26].

### 4.4. Statistical Analysis

Data were collected and analyzed using SPSS (version 26; Chicago, IL, USA). Descriptive statistics included counts and percentages for qualitative variables, mean and standard deviation (SD) for normally distributed continuous variables, or median and interquartile range (IQR) for non-parametric variables. For comparison of qualitative data, Chi-square tests or Fischer exact tests were used as appropriate. For continuous data, the Student’s *t*-test or Analysis of Variance (ANOVA) was used in the case of parametric data, and the Mann–Whitney U test or Kruskal–Wallis test was used in the case of non-parametric data. A *p*-value < 0.05 was considered statistically significant.

## 5. Conclusions

This study reports that in the sub-Saharan African context, Gram-negative bacteria are the most common causative microorganisms of FRI, in contrast to high-resource settings. In early FRI, which represents more than half of cases in this context, infections are mostly polymicrobial, involving Gram-negative bacteria. Late FRI is mostly monomicrobial, with Gram-positive bacteria being more frequent. Although *Staphylococcus aureus* remains the most frequently isolated microorganism in FRI, its proportion is lower than in the series from HICs. This study revealed striking resistance rates to commonly used antibiotics, which requires urgent action to limit the rise in antibiotic resistance worldwide. This study provides a basis for the guidance of EAT for FRI in a sub-Saharan African context. We recommend the combination of vancomycin and amikacin or vancomycin and imipenem as the initial systemic treatment of FRI, as it is the most effective empirical antibiotic therapy with locally available antibiotics. Large multicenter prospective studies are needed to investigate the association between clinical presentation/onset of infection, causative microorganisms, susceptibility profile, management, and outcome.

## Figures and Tables

**Table 1:** Patient and FRI characteristics.

Variable	Value (N = 217)
Age (in years) (mean ± SD, range)	40.64 ± 15.12 (5–85)
Gender: number of men (%)	150 (69.1%)
Body mass index (kg/m^2^)	27.22 ± 4.59
Alcohol, number (%)	14 (6.5%)
Obesity, number (%)	7 (4.8%)
Diabetes, number (%)	6 (2.7%)
HIV, number (%)	3 (1.4%)
Smoking, number (%)	5 (2.3%)
Initial fracture	
Closed fracture	110 (52.1%)
Open fracture	107 (47.9%)
Site of the FRI	
Tibia	95 (42.6%)
Femur	87 (39.0%)
Ankle	13 (5.8%)
Humerus	8 (3.6%)
Radius	7 (3.1%)
Ulna	6 (2.7%)
Fibula	3 (1.3%)
Carpus	2 (0.9%)
Tarsus	1 (0.5%)
Patella	1 (0.5%)
Classification of FRI	
Early	115 (53.0%)
Delayed	37 (17.1%)
Late	65 (29.9%)
Number of isolated microorganisms per culture	
0	37 (15.1%)
1	100 (40.6%)
2	67 (27.2%)
3	39 (15.9%)
4	3 (1.2%)

**Table 2:** Profile of identified Organisms.

Microorganism	Number	Percentage of Positive Cultures
***Gram-positive bacteria***		
*Staphylococcus aureus*	*69*	*19*
*Coagulase-negative Staphylococcus*	*21*	*5.8*
*Enterococcus faecalis*	*16*	*4.4*
*Enterococcus faecium*	*3*	*0.8*
*Other Enterococcus*	*6*	*1.6*
*Group A Streptococcus*	*4*	*1.1*
*Other Streptococcus*	*4*	*1.1*
*Other GPC*	*1*	*0.3*
***Gram-negative bacteria***		
***Enterobacterales***		
*Enterobacter cloacae*	43	11.8
*Klebsiella pneumoniae*	36	9.9
*Escherichia coli*	35	9.6
*Proteus mirabilis*	17	4.7
*Morganella morganii*	14	3.9
*Providencia stuartii*	8	2.2
*Citrobacter freundii*	5	1.4
*Other Enterobacterales*	*30*	*8.3*
***Non-fermenting Gram-negative bacilli***		
*Pseudomonas aeruginosa*	27	7.4
*Acinetobacter baumannii*	4	1.1
*Sternotrophomonas maltophilia*	2	0.6
*Other non-fermenting Gram-negative bacilli*	11	3.1
***Other GNB***	6	1.6
Total	363	100%

GNB; Gram-negative bacteria, GPC; Gram-positive cocci.

**Table 3:** Comparison between early, delayed, and late FRI.

Variable	Early FR In (%)	Delayed FR In (%)	Late FR In (%)	Whole Group n (%)	*p*-Value *
Number of cases	127 (51.6)	44 (17.9)	75 (30.5)	246 (100)	
**Culture type**					
Monomicrobial (%)	45 (35.4)	18 (41.9)	37 (48.7)	100 (40.6)	0.174
Polymicrobial (%)	71 (55.9)	18 (41.9)	20 (27.6)	109 (44.3)	**0.000**
Culture-negative (%)	11 (8.7)	8 (18.6)	18 (23.7)	37 (15.1)	**0.012**
**Gram stain**					
Gram-negative (%)	156 (70.9)	41 (73.2)	42 (48.3)	239 (65.8)	**0.000**
Gram-positive (%)	64 (29.1)	15 (26.8)	45 (51.7)	124 (34.2)	**0.000**
**Species isolated**					
*Staphylococcus aureus*	33 (15)	7 (12.5)	29 (33.3)	69 (19)	**0.000**
*Enterobacter cloacae*	23 (10.5)	12 (21.4)	8 (9.2)	43 (11.8)	0.052
*Klebsiella pneumonia*	25 (11.4)	3 (5.4)	8 (9.2)	36 (9.6)	0.393
*Escherichia coli*	22 (10)	8 (14.3)	5 (5.7)	35 (9.6)	0.231
*Pseudomonas aeruginosa*	18 (8.2)	4 (7.1)	5 (5.7)	27 (7.4)	0.761
*CoNS*	8 (3.6)	6 (10.7)	7 (8)	21 (5.9)	0.075
*Proteus mirabilis*	10 (4.5)	4 (7.1)	3 (3.4)	17 (4.7)	0.587
*Enterococcus faecalis*	13 (5.9)	-	3 (3.4)	16 (4.4)	0.139
*Morganella morganii*	7 (3.2)	3 (5.4)	4 (4.6)	14 (3.9)	0.691
Others	61 (27.7)	9 (16.1)	15 (17.2)	85 (23.7)	0.055
Total	220 (100)	56 (100)	87 (100)	363 (100)	

* *p* values in bold are statistically significant at the 0.05 level.

**Table 4:** A comparison of the microbiological profile between this study and the recently published series.

	This Study	Ferriera et al. [13]	Corrigan et al. [20]	Baertl et al. [19]	Depypere et al. [18]	Patel et al. [3]
**Location**	Cameroon	South Africa	UK and The Netherlands	Germany	Belgium	UK
**Number**	246	267	433	117	194	325
**Time of Presentation (%)**						
Early	52	NR	12	16	18	NR
Delayed	17	NR	19	51	38	NR
Late	31	NR	69	33	44	NR
**Type of Culture**						
Polymicrobial (%)	44.3	14	36	8.6	25.3	34.2
Monomicrobial (%)	40.6	67	46	82	71.1	48.3
Culture-negative (%)	15.1	19	19	9.4	3.6	24.4
**Gram Negatives (%)**	65.8	55	22	16.4	21.1	39.7
***Staphylococcus Aureus* (%)**	19	27	31	40	31.4	24.4

## Data Availability

Data are available from the corresponding author on reasonable request.
